# Supplementary material for: Association of pulmonary artery bifurcation angle shift with contralateral stenosis after post-arterial switch in the pediatric transposition of great artery patients
Source: PLoS One. 2025 Jun 20;20(6):e0325206. doi: 10.1371/journal.pone.0325206 (PMC12180649; doi:10.1371/journal.pone.0325206)
Supplement: Attachment — Submitted filename: Point-by-Point Respose to reviewers.docx [file pone.0325206.s002.docx]

**Point-by-point responses to reviewer’ comment**

**The association between a greater rightward or leftward shift of the pulmonary artery bifurcation after the arterial switch operation and contralateral branch pulmonary artery stenosis in the pediatric transposition of great artery patients**

Thank you very much for your kind considering of our manuscript. We appreciate the constructive and very helpful feedback and the supportive comments from the reviewers. We have carefully considered the reviewers’ comments and suggestions and have edited the manuscript to address them. Based on those suggestions, we believe the manuscript has greatly improved after the revision.

We have addressed each comment as outlined below with the yellow color highlight in the manuscript.

**Reviewer #1: Your manuscript is commendable for its clarity and novelty. To enhance its impact, I recommend:**

**1.Expanding the clinical implications in both the discussion and conclusion sections**

**Answer:** We would like to thank the reviewer for this suggestion. We expanded the clinical implication in both the discussion and the conclusion sections.

Discussion section

These findings may indicate the need for refinements in pre-ASO LeCompte surgical techniques, particularly in planning and adjusting the angle between AAO and PA bifurcation to less than 20 degrees.

Conclusion section

Pre-ASO LeCompte strategies to optimize the positioning and angulation of pulmonary artery branches may improve the outcome of surgery.

**Discussion section: Pages 22, lines 352-354**

**Conclusion section: Pages 23, lines 379-380**

**2. Improving the visual data representation to ensure clarity and accessibility for readers. (try taking plot exports in a higher quality)**

**Answer:** We would like to thank the reviewer for this suggestion. We replaced a figure with higher-quality images.

The revised Figure 3 are shown below.

**Reviewer #2: Dear Authors,
I found your manuscript interesting, for better illustration, I have some suggestion:**

**1.it should be better explain more comprehensively the way of measuring the angle between AAO and PA bifurcation, and also mention the parameters that lead to measurement error.**

**Answer:** We would like to thank the reviewer for this suggestion. We explain how to measure the angle between AAO and PA bifurcation. We mention the parameters of the measurement of the angle between AAO an PA bifurcation that can lead to measurement error in the limitation section.

Material and methods section

“Pulmonary artery bifurcation to ascending aortic angle is defined as the angle between two lines: one line extends from the center of the ascending aorta to the pulmonary artery bifurcation, while the other is a reference line drawn from the spinous process to the anterior spinal border convexity. To measure this angle, the axial images are first scrolled through to locate the slice showing the tip of the reverse V-shaped pulmonary arterial bifurcation. A line is then drawn from the tip of the pulmonary arterial bifurcation to the center of the ascending aorta. The reference vertical line is drawn from the tip of the spinous process to the anterior most point of the spinal body convexity. This reference line should pass through the center of the spinal canal and intersect both the anterior border convexity and posterior border of the spinal canal. In cases of vertebral deformity, this line is drawn using the average position between these anatomical markers.”

Study limitation

“Second, measurement errors in the angle between the AAO and PA bifurcation may arise due to vertebral deformities (e.g., scoliosis or butterfly vertebrae) and inconsistent selection of the key image for pulmonary artery bifurcation. However, scrolling through image stacks helps identify the most representative bifurcation image.”

**Material and methods section: Page 8, line2 148-158**

**Study limitation: Page 22 lines 362-366**

**2.explaining about two modality imaging (CCT, CMR) difference in evaluation of this angle.**

**Answer:** In considering the differences between CCT and CMR, these angles rely on bony landmarks for measurement. With CMR imaging, obtaining an accurate vertical reference line may be challenging because bony landmarks may not be as well visualized as on the CCT due to the inherent characteristics of MR imaging.

**3.explain your suggestion to the surgeon that how can apply this issue for alleviation PAB stenosis complication.**

**Answer:** We would like to thank the reviewer for this suggestion. We suggest to the surgeon how to apply this issue for alleviation of branch pulmonary arteries stenosis complication

“These findings may indicate the need for refinements in pre-ASO LeCompte surgical techniques, particularly in planning and adjusting the angle between AAO and PA bifurcation to less than 20 degrees.”

**Page22 lines 352-354**

**Reviewer #3: This manuscript provides relevant and compelling data and findings on the clinical significance of the pulmonary artery bifurcation angle. Below are some comments and suggestions that the authors could consider expanding on and clarifying in the paper.**

**1. Abstract states % without raw numbers.**

**Answer:** We would like to thank the reviewer for this suggestion. We add raw data number in the abstract.

Of the thirty patients enrolled in this study, 46.67% (14/30) had an isolated branch pulmonary artery stenosis, and 53.33% (16/30) had no pulmonary artery stenosis.

**Page2 lines 34-36**

**2. Direct evidence for angulation-stenosis hypothesis?**

Answer: We would like to thank the reviewer for this question. To the best of our knowledge, there is no publication showing the correlation of pulmonary artery bifurcation to ascending aortic angle and isolated branch PA stenosis. Morgan CT et al. showed that neo-pulmonary to neo-aortic geometry impacts LPA size and perfusion of the left lung. Allen H et.al. showed that the branch pulmonary stenosis likely arises from multiple causes, with post-ASO obstruction potentially resulting from the pulmonary elongated and stretched pathway.

References

1. Morgan CT, Mertens L, Grotenhuis H, Yoo SJ, Seed M, Grosse-Wortmann L. Understanding the mechanism for branch pulmonary artery stenosis after the arterial switch operation for transposition of the great arteries. Eur Heart J Cardiovasc Imaging. 2017 Feb;18(2):180-185. doi: 10.1093/ehjci/jew046. Epub 2016 Mar 29. PMID: 27025515.
2. Allen H, Driscoll DJ, Shaddy RE, Feltes TF. Moss and Adams Heart Disease in Infants, Children, and Adolescents: Including the Fetus and Young Adult. 8th ed. Philadelphia: Wolters Kluwer Health/Lippincott Williams & Wilkins; 2013. p1792.
3. **The introduction could more clearly articulate the importance of the angle of pulmonary artery bifurcation in post-ASO stenosis and anatomical aspects of the pulmonary arteries post-ASO and how these relate to stenosis.**

**Answer:** We would like to thank the reviewer for this suggestion. In the introduction, we added the important hypothesis in post-ASO stenosis and anatomical aspects of the pulmonary arteries post-ASO.

**“**According to a previous study, geometric anatomical factors contributing to branch pulmonary stenosis likely have multiple causes, with post-ASO branch pulmonary stenosis potentially arising from a lengthened and tensioned pulmonary artery pathway”

“We hypothesize the new geometric anatomy orientations, including the angle between AAO and PA bifurcation after surgery are the risk factors of branch PA stenosis.”

**Page 4, lines 68-70**

**Page 5, lines 78-79**

4. **The study’s retrospective design introduces selection bias, particularly with exclusions of complex cases. This limits generalizability to the broader TGA population.
   Justification for CCT timing could be stronger. The rationale for performing CCT specifically after ASO? Was there a specific clinical context for ordering CCT in these patients, or was it part of a routine follow-up protocol?**

**Answer:** We would like to thank the reviewer for this suggestion. Due to the retrospective study, we didn’t have a protocol or routine for follow-up post-op ASO at that time. We explored the specific clinical conditions for ordering CCT in more detail. We found that the median age of CCT was 7.8 years old. The clinical condition for ordering CCT was the limitations of echocardiographic imaging that made it difficult to assess the pulmonary arteries conclusively; obtaining branch PA Doppler measurements was technically unfeasible because of echocardiographic limitations and an improper Doppler ultrasound angle. Pediatric cardiologists suspected branch PA stenosis based on the presence of a heart murmur. We discuss this in more detail in the discussion section.

“With excellent inter-rater reliability, our findings highlight the utility of advanced cardiac imaging, including CCT and CMR, is valuable for evaluating post-ASO patients, especially in patients over five years post-surgery or those with echocardiographic limitations or abnormal heart murmurs. We suggest long-term surveillance of TGA patients following ASO surgery, particularly beyond five years, using advanced cardiac imaging to enable early detection of abnormalities and ensure timely planning for appropriate management, including intervention or surgery.”

**Page 21, lines 332-337**

5. **Briefly explain justification for using the extended Wilcoxon rank-sum test by Cuzick for trend analysis across the three stenosis groups.**

**Answer:** Thank you for your feedback. In certain situations, slope estimates, along with their corresponding confidence intervals, can provide more meaningful insights than hypothesis tests assessing a zero slope [1]. We hypothesise that there is an ordered relationship between the new angle and the categories of Left PA stenosis, No PA stenosis, and Right PA stenosis.

An extension of the Wilcoxon rank-sum trend test has been developed to accommodate data where a variable is measured across three or more ordered groups. Furthermore, this trend test demonstrates greater statistical power as it captures a more precise confidence interval [2].

**Reference**

1. Lui KJ, Cumberland WG. A Wilcoxon-type test for trend. *Stat Med*. 1995;14(4):445-446. doi:10.1002/sim.4780140409
2. Cuzick J. A Wilcoxon-type test for trend. Stat Med. 1985;4(1):87-90.
6. **30 patients, with only 7 per subgroup (RPA/LPA stenosis), reduce statistical power. Table 3 reports non-significant differences in neo-aortic root size (p=0.591) but may reflect Type II error.**

Answer: Thank you for your feedback. The statistical analysis was performed between the isolated stenosis group (N = 14) and the non-stenosis group (N = 16). While we recognise the small sample size, this limitation has been acknowledged.

“Lastly, the study population had a small sample size. A multicentre study with a larger patient cohort and a well-standardised long-term surveillance protocol for post-ASO patients using advanced cardiac imaging is warranted.”

**Page 2, lines 367-369**

7. **Table 2 reports p=0.019 for gestational age but tests 10+ variables. No correction increases type I error.**
8. **Table 2 shows a statistical difference in birth weight and gestational age between the groups at baseline. Multivariable analysis to adjust for these factors could better clarify the association between the pulmonary artery bifurcation angle and stenosis.**

**Answers for no7-8:**  We appreciate the opportunity to address your concerns. We agree that without correction, the probability of a statistically significant outcome occurring by chance is increased. However, the primary objective of our study is to explore the possible mechanisms of branch PA stenosis using CCT parameters. Therefore, adjusting the P-value for these factors may not be a primary concern in this context.

Regardless of whether the correction leads to rejecting the null hypothesis or not, as you have suggested, multivariable analysis adjusted for gestational age, birth weight, or sex should be conducted when there is a biologically plausible.

We have edited the method accordingly in line 215-217:

“The linear trend between the angles and the PA proximal-distal ratio was analyzed using multivariable linear regression adjusted by age gestational age birthweight and sex.”

**Page 11, line 215-217**

The revised Figure 3 are shown below.

The association between the pulmonary artery bifurcation-to-ascending aorta angle in patients with right PA stenosis and those without PA stenosis has changed to a minimal effect and is no longer statistically significant. All other associations remain consistent with the unadjusted trend.

9. **The manuscript does not clarify how outliers or ambiguous CCT measurements were resolved between raters.**

Answer: Thank you for your feedback. We apologise for not providing more details on this issue. In cases where the ICC demonstrates poor reliability, the selection of value between the two raters will be determined based on the opinion of a third expert who was not involved in this study.

We have added the method accordingly in line 210-213: “If both angles showed good or excellent inter-rater reliability, we randomly selected values from one rater for further assessment of the angle properties. In cases where the ICC demonstrates poor reliability, the selection of value between the two raters will be determined based on the opinion of a third expert who was not involved in this study.”

**Page 11, lines 210-213**

10. **The manuscript states 14 patients had isolated branch PA stenosis, 7 RPA and 7 LPA cases. Table 3 (isolated PA branch stenosis N=14) reports 7 cases, 4 RPA (28.6%) and 3 LPA (21.4%). This leaves 7 unaccounted cases.**

**Answer:** Thank you for your feedback. We apologized for the confusion in our manuscript. The mentioned results aim to highlight inaccuracies in echocardiographic findings. Approximately 50% of patients with isolated PA stenosis were underdiagnosed by echocardiography. We have stated this finding in **lines 277-278** as follow:

“In this study, echocardiography detected the pulmonary artery branch stenosis in only 50% of the cases.”

And in the discussion in **lines 326-329** as follows:

“Echocardiography detected pulmonary artery stenosis around 50% of the time at follow-up. Echocardiography's limitation in identifying lesions outside the heart could delay intervention or surgery treatment (20). Doppler measurements of the branch PA were technically unfeasible in 20% of patients from previous studies (21, 22).”

**Result section, Page 15, lines 277-278**

**Discussion section, Pages 20-21, line326-329**

11. **Does the result section report the calculated ICC values to support 'Both angles demonstrated excellent inter-rater reliability' in the discussion?**

**Answer**: Thank you for your suggestions. We have added a discussion on this issue in line 336-342 as follow “With excellent inter-rater reliability, our findings highlight the utility of advanced cardiac imaging, including CCT and CMR, is valuable for evaluating post-ASO patients, especially in patients over five years post-surgery or those with echocardiographic limitations or abnormal heart murmurs. We suggest long-term surveillance of TGA patients following ASO surgery, particularly beyond five years, using advanced cardiac imaging to enable early detection of abnormalities and ensure timely planning for appropriate management, including intervention or surgery.”

12. **Directly mentioning the p-values for the trend analysis in the figure 3 captions would make the figure clearer and self-contained.**

**Answer:** Thank you for your suggestions. We have provided the p-value on each linear adjusted trend within the legend and figure caption as shown below.

**“****Fig 3. Angles Distribution in Isolated Branch Pulmonary Artery Stenosis** illustrates a Box plots (A) Neo-pulmonary artery to neo-aorta angle and (B) Pulmonary artery bifurcation to ascending aorta angle, with linear adjusted trends for PA proximal distal ratio (C, D) in right PA stenosis (navy), no PA stenosis (bluish grey), and left PA stenosis (red). In (B), no overlap is observed between subgroups. Dashed red lines mark angle thresholds (-15 and 15 degrees), while in (C, D), the horizontal dashed red line indicates a PA stenosis threshold of 0.75. Linear trend P-values for left and right PA stenosis vs no PA stenosis: (C) 0.506 and 0.932; (D) 0.011 and 0.269, respectively.”

13. **Justification for figure 3 '-15/15 degrees' cutoffs?**

**Answer:** This is the first study to report the use of this technique in Thai patients, and no established cutoff points currently exist. We propose this cutoff based on our data to illustrate the normal range observed in our study population with favorable outcomes (no PA stenosis).

14. **The conclusion suggests 'pre-ASO surgical strategy changes' but lacks evidence that angulation modification improves outcomes.**

**Answer:** We would like to thank the reviewer for this suggestion. We expanded the clinical implication in both the discussion and the conclusion sections.

Discussion section

These findings may indicate the need for refinements in pre-ASO LeCompte surgical techniques, particularly in planning and adjusting the angle between AAO and PA bifurcation to less than 20 degrees.

Conclusion section

Pre-ASO LeCompte strategies to optimize the positioning and angulation of pulmonary artery branches may improve the outcome of surgery.

Page 21

15. **The 'over-stretching' hypothesis lacks direct evidence. The proposed mechanism as the cause of stenosis due to angle variations lacks strong supporting evidence.**

**Answer:** We would like to thank the reviewer for this comment. We explain in detail the mechanism as the cause of stenosis.

“The stretched, elongated, and tensioned pulmonary artery course may contribute to the mechanism underlying branch pulmonary stenosis.”

Reference: Tang T, Chiu IS, Chen HC, Cheng KY, Chen SJ. Comparison of pulmonary arterial flow phenomena in spiral and Lecompte models by computational fluid dynamics. J Thorac Cardiovasc Surg. 2001;122(3):529-34.

**Page 21, line 347-348**

16. **While the study identifies an association between the pulmonary artery bifurcation angle and post-ASO stenosis, the clinical actionability of this finding is not clearly established**

**Answer:** We would like to thank the reviewer for this suggestion. We explain the clinical implication in both the discussion and conclusion sections.

Discussion section

These findings may indicate the need for refinements in pre-ASO LeCompte surgical techniques, particularly in planning and adjusting the angle between AAO and PA bifurcation to less than 20 degrees.

Conclusion section

Pre-ASO LeCompte strategies to optimize the positioning and angulation of pulmonary artery branches may improve the outcome of surgery.

**Discussion section: Pages 22, lines 352-354**

**Conclusion section: Pages 23, lines 379-380**

17. **Limitations in design need to be acknowledged in the discussion more explicitly and briefly in the abstract.**

**Answer:** Thank you for your suggestions. We have edited according to your suggest in discussion section as follow**:**

**“**First, as an observational retrospective study, it is subject to inherent drawbacks, including data quality issues and potential selection bias. The selection of cases for advanced imaging may have been influenced by echocardiographic limitations, such as technical constraints and suboptimal Doppler ultrasound angles, making pulmonary artery assessment challenging. Additionally, pediatric cardiologists may have selected cases based on heart murmurs, potentially increasing the observed incidence of PA stenosis in our cohort. Second, measurement errors in the angle between the AAO and PA bifurcation may arise due to vertebral deformities (e.g., scoliosis or butterfly vertebrae) and inconsistent selection of the key image for pulmonary artery bifurcation. However, scrolling through image stacks helps identify the most representative bifurcation image. Third, this study was performed at a single center, potentially limiting its generalizability. Lastly, the study population had a small sample size. A multicentre study with a larger patient cohort and a well-standardised long-term surveillance protocol for post-ASO patients using advanced cardiac imaging is warranted”.

And in abstract in line 44-45 as follow:

“Pulmonary artery bifurcation angle shifts may contribute to isolated branch pulmonary stenosis. Pre-operative strategies by planning and adjusting the angle between AAO and PA bifurcation should be less than ±20 degrees. A larger study incorporating advanced cardiac imaging into routine ASO follow-up is warranted to confirm our findings.”

**Study limitation section: Pages 22, lines 357-369**

**Abstract section: Pages 2, lines 42-45**

18. **Expand challenges and future research directions**

**Answer:** We would like to thank the reviewer for this suggestion. We address future research directions in study limitation part.

“Future research utilizing 4D flow technology by cardiac MRI for advanced hemodynamic analysis, including wall shear stress and energy loss, may provide new insights into the underlying mechanisms of branch pulmonary artery stenosis. Preoperative planning with computational modeling using computational fluid dynamics may improve surgical strategies to optimize hemodynamics and enhance surgical outcomes.”

**Page 22-23, lines 370-374**

**Reviewer #4: This manuscript investigates the association between changes in pulmonary artery bifurcation angles and isolated branch pulmonary artery stenosis following the ASO in pediatric patients with TGA. The study provides valuable insights by utilizing cardiac CT imaging to identify geometric alterations potentially contributing to post-operative complications. The findings are significant for improving surgical strategies and post-operative surveillance. However, the manuscript requires improvements in clarity, structure, and depth of discussion to better support its conclusions and enhance reader comprehension. Below are detailed comments for revisions:**

**1. The title is informative but long.**

**Answer:** We would like to thank the reviewer for this suggestion. We changed the title as per your suggestion.

“Association of Pulmonary Artery Bifurcation Angle Shift with Contralateral Stenosis after Post-arterial Switch in the Pediatric Transposition of Great Artery Patients”

**Page 1, lines 1-2**

**2. Page 2, Line 10: Define “arterial switch operation” clearly**

**Answer:** We would like to thank the reviewer for this suggestion. We defined arterial switch more clearly

“The operation of choice is the arterial switch operation (ASO), which reestablishes normal blood flow and proper alignment of the ventricles and great arteries. It involves transecting the aorta and pulmonary arteries above the sinuses and attaching them to their correct ventricles. The pulmonary arteries are moved anteriorly to the aorta using the LeCompte maneuver, while the coronary arteries are detached and implanted onto the neo-aorta”

**Page 4, lines 56-60**

**3. Page 2, Line 33: Expand "The mechanism of branch stenosis is still unclear" by mentioning prior hypotheses or research gaps to highlight the novelty of your study.**

**Answer:** We would like to thank the reviewer for this suggestion. We mention the hypotheses to highlight the novelty of our study.

“According to a previous study, geometric anatomical factors contributing to branch pulmonary stenosis likely have multiple causes, with post-ASO branch pulmonary stenosis potentially arising from a lengthened and tensioned pulmonary artery pathway”

“We hypothesize the new geometric anatomy orientations, including the angle between AAO and PA bifurcation after surgery are the risk factors of branch PA stenosis”.

**Page 4, lines 68-70**

**Page 5, lines 78-79**

**4. Page 7, Table 1: Add total percentages for categories such as "sex" and "TGA type" for better interpretation.**

**Answer:** Thank you for your suggestion. We have made the necessary edits accordingly**.**

**5. Page 8, Line 25: Briefly explain "Balloon atrial septostomy" for readers unfamiliar with this procedure.**

**Answer:** We would like to thank the reviewer for this suggestion. We explain “balloon atrial septostomy” in the definition part.

“Balloon atrial septostomy is a catheter-based procedure in which a wire is threaded through the atrial septum. A balloon is inflated in the left atrium and pulled back into the right atrium, creating an opening to enhance blood mixing.”

Ref: Haeffele C, Lui GK. Dextro-Transposition of the Great Arteries: Long-term Sequelae of Atrial and Arterial Switch. Cardiol Clin. 2015;33(4):543-58, viii.

**Page 10, lines195-197**

**6. Figure 2: Labeling for angles in Figures 2C and 2F is difficult to follow. Clearer labels or additional descriptions in the legend would improve comprehension.**

**Answer:** We would like to thank the reviewer for this suggestion. We addressed these in greater detail as per your suggestion.

**Fig 2.** Great Vessel Anatomy and Angular Measurements Following the LeCompte Maneuver. 2A: Great vessel anatomy in an axial plane. Right pulmonary artery stenosis is defined when the ratio of proximal to distal measurements is <0.75. 2B: Volume-rendered 3D reconstructions of a TGA patient after ASO with LeCompte maneuver, demonstrating RPA stenosis. 2C: Neo-pulmonary to neo-aortic angle (alpha angle). This angle is measured between a line through the centers of the neo-aortic and neo-pulmonary roots and a reference vertical line. For graphic demonstration, a parallel line to the reference vertical line is drawn. A rightward deviation results in a negative alpha angle. 2D: Neo-pulmonary to neo-aortic angle (alpha angle). This angle is measured between a line through the centers of the neo-aortic and neo-pulmonary roots and a reference vertical line. A leftward deviation results in a positive alpha angle. 2E: Pulmonary artery bifurcation to ascending aortic angle (beta angle). This angle is defined between a reference vertical line and a line from the pulmonary artery bifurcation to the center of the ascending aorta (AAO). A rightward deviation results in a negative beta angle. 2F: Pulmonary artery bifurcation to ascending aortic angle (beta angle). This angle is defined between a reference vertical line and a line from the pulmonary artery bifurcation to the center of the ascending aorta (AAO). A leftward deviation results in a positive beta angle.

**Page 7 -8, lines127-142**

**7. Page 14, Figure 3: Ensure figure legends are detailed enough to be understood without referring back to the text.**

**Answer:** Thank you for your suggestions. We have provided figure legends as your suggestion

**“Fig 3. Angles Distribution in Isolated Branch Pulmonary Artery Stenosis** illustrates a Box plots (A) Neo-pulmonary artery to neo-aorta angle and (B) Pulmonary artery bifurcation to ascending aorta angle, with linear adjusted trends for PA proximal distal ratio (C, D) in right PA stenosis (navy), no PA stenosis (bluish grey), and left PA stenosis (red). In (B), no overlap is observed between subgroups. Dashed red lines mark angle thresholds (-15 and 15 degrees), while in (C, D), the horizontal dashed red line indicates a PA stenosis threshold of 0.75. Linear trend P-values for left and right PA stenosis vs no PA stenosis: (C) 0.506 and 0.932; (D) 0.011 and 0.269, respectively.”

**8. Page 20, Line 15: Simplify "The proposed explanation for this occurrence is a more abnormal rightward or leftward..."**

**Answer:** We would like to thank the reviewer for this suggestion. We explain in more detail.

“The stretched, elongated, and tensioned pulmonary artery course may contribute to the mechanism underlying branch pulmonary stenosis. The proposed explanation for this occurrence is an abnormally greater rightward or leftward angulation of the PA, which may result in blood from the right ventricle being preferentially diverted to the other side of the PA. This may cause the abnormal velocity profile and wall-shear stress distribution in isolated branch PA stenosis”

**Page 21-22, lines 347-352**
 **9. Page 22, Line 3: Expand on how selection bias may have influenced your findings and provide suggestions for mitigating this issue in future studies.**

**Answer:** We would like to thank the reviewer for this suggestion. We expand on how selection bias may influence our findings.

“First, as an observational retrospective study, it is subject to inherent drawbacks, including data quality issues and potential selection bias. The selection of cases for advanced imaging may have been influenced by echocardiographic limitations, such as technical constraints and suboptimal Doppler ultrasound angles, making pulmonary artery assessment challenging. Additionally, pediatric cardiologists may have selected cases based on heart murmurs, potentially increasing the observed incidence of PA stenosis in our cohort”

“Lastly, the study population had a small sample size. A multicentre study with a larger patient cohort and a well-standardised long-term surveillance protocol for post-ASO patients using advanced cardiac imaging is warranted”

**Page 22, lines 357-362, lines367-369**

**10. Abstract: Consider including more specific results to highlight the study's key findings, such as precise angle measurements.**

**Answer:** We would like to thank the reviewer for this suggestion. We include more specific results as per your suggestion

“Pulmonary artery bifurcation angle shifts may contribute to isolated branch pulmonary stenosis. Pre-operative strategies should plan to adjust the angle between AAO and PA bifurcation to less than 20 degrees. Incorporating advanced cardiac imaging into routine ASO follow-up is warranted”

**Abstract part: Page2, lines 42-45**

**11. Discussion: The proposed mechanisms of over-stretching should be further elaborated and supported with literature evidence.**

**Answer:** We would like to thank the reviewer for this suggestion. We discussed in more detail in **the discussion part.**

“The stretched, elongated, and tensioned pulmonary artery course may contribute to the mechanism underlying branch pulmonary stenosis. The proposed explanation for this occurrence is an abnormally rightward or leftward angulation of the PA, which may result in blood from the right ventricle being preferentially diverted to the other side of the PA. This may cause the abnormal velocity profile and wall-shear stress distribution in isolated branch PA stenosis.”

**Page 21,22, lines 347-352**
