# Supplementary material for: Association of pulmonary artery bifurcation angle shift with contralateral stenosis after post-arterial switch in the pediatric transposition of great artery patients
Source: PLoS One. 2025 Jun 20;20(6):e0325206. doi: 10.1371/journal.pone.0325206 (PMC12180649; doi:10.1371/journal.pone.0325206)
Supplement: Attachment — Submitted filename: Point-by-Point_Respose_to_reviewers_auresp_2.docx [file pone.0325206.s003.docx]

**Association of Pulmonary Artery Bifurcation Angle Shift with Contralateral Stenosis after Post-arterial Switch in the Pediatric Transposition of Great Artery Patients**

**Old Title: The association between a greater rightward or leftward shift of the pulmonary artery bifurcation after the arterial switch operation and contralateral branch pulmonary artery stenosis in the pediatric transposition of great artery patients**

Thank you very much for your kind consideration of our manuscript. We appreciate the constructive and very helpful feedback and the supportive comments from the reviewers. We have carefully considered the reviewers’ comments and suggestions and have edited the manuscript to address them. Based on those suggestions, we believe the manuscript has greatly improved after the revision.

**Revision 1**

We have addressed each comment as outlined below with the yellow color highlight in the manuscript.

**Revision 2**

We have addressed each comment as outlined below with the green color highlight in the manuscript.

**#Additional Editor Comments:** Congratulations on your excellent paper. Consider adding an abbreviation table at the beginning of the manuscript for clarity. The abstract should follow a structured format, and the results section should be improved to better represent the study's findings. In line 53, the statement “The prevalence of TGA is between 20-30 per 100,000 live births” needs more context—please specify the location and time period. Additionally, extract the approval date from the text and include it in the cover letter or title page.

**Answer:**

- **Comment 1:** Consider adding an abbreviation table at the beginning of the manuscript for clarity.
  - **Response:** Thank you for the suggestion. We have now included an **Abbreviation Table** at the beginning of the manuscript, listing all relevant terms used for better clarity and ease of reading.
  - Page 4
- **Comment 2:** The abstract should follow a structured format, and the results section should be improved to better represent the study's findings.
  - **Response:** We appreciate this recommendation. The abstract has been **revised into a structured format** with the following headings: **Background, Methods, Results, and Conclusion,** in accordance with PLOS ONE guidelines. We also rewrote the result section in the abstract part to better represent the study's findings.
  - Page 2-3 lines 26-47
- **Comment 3:** In line 53, the statement “The prevalence of TGA is between 20-30 per 100,000 live births” needs more context
  - **Response:** Thank you for pointing this out. We have revised the sentence for clarity and context. It now reads:
    “The prevalence of TGA is estimated to be between 20 and 30 per 100,000 live births, based on population-based studies conducted in Europe and North America from 2000 to 2010.” A corresponding reference has been added.
  - **Reference:** **van der Linde D, Konings EE, Slager MA, et al.** Birth prevalence of congenital heart disease worldwide: a systematic review and meta-analysis. J Am Coll Cardiol. 2011;58(21):2241-2247. doi:10.1016/j.jacc.2011.08.025
  - Page 5 line 71-73
- **Comment 4:** Please specify the location and time period. Additionally, extract the approval date from the text and include it in the cover letter or title page.
  - **Response:** The Institutional Review Board of the Faculty of Medicine, Chiang Mai University, Chiang Mai, Thailand, has been extracted from the Methods section and is now included in the cover letter.
  - The study was approved by the Institutional Review Board of the Faculty of Medicine, Chiang Mai University, Chiang Mai, Thailand, on 16/11/2022 (approval no. 411/2022, date of approval 16/11/2022, and date of expiration 15/11/2023).
  - Page 6-7 lines 111-120
  - Cover letter

**# Reviewers' comments:**

2. Is the manuscript technically sound, and do the data support the conclusions?

The manuscript must describe a technically sound piece of scientific research with data that supports the conclusions. Experiments must have been conducted rigorously, with appropriate controls, replication, and sample sizes. The conclusions must be drawn appropriately based on the data presented.

**Answer:** We would like to thank reviewers to this comment. The **conclusions** are directly supported by the data, particularly the observed relationship between PA bifurcation angles and isolated branch pulmonary artery stenosis and are framed within the context of the study’s limitations.

“Alterations in the PA bifurcation angle relative to the AAO may contribute to the development of isolated branch PA stenosis following ASO. Preoperative planning to optimize the PA bifurcation angle to within ±20 degrees may help reduce the risk of postoperative stenosis. A larger study incorporating advanced cardiac imaging into routine ASO follow-up is warranted.”

Page 2-3 line 44-47

4. The [PLOS Data policy](https://apac01.safelinks.protection.outlook.com/?url=http%3A%2F%2Ftrack.editorialmanager.com%2FCL0%2Fhttp%3A%252F%252Fwww.plosone.org%252Fstatic%252Fpolicies.action%2523sharing%2F1%2F010f0195eed6cf29-d84e1d5e-2b8b-455e-a9ab-3b24cdfc391b-000000%2Fo4LHvG3bPBZ4X-kszCtrh3DSvva_qgP1QdOTEl3OAMc%3D204&data=05%7C02%7C%7Cfd99bdd0137f48a43e3b08dd70b79f37%7C84df9e7fe9f640afb435aaaaaaaaaaaa%7C1%7C0%7C638790656148228611%7CUnknown%7CTWFpbGZsb3d8eyJFbXB0eU1hcGkiOnRydWUsIlYiOiIwLjAuMDAwMCIsIlAiOiJXaW4zMiIsIkFOIjoiTWFpbCIsIldUIjoyfQ%3D%3D%7C0%7C%7C%7C&sdata=L5nn%2FUswR12AtBPXdngfK5D298pSyHV6jAVHoUWrTrs%3D&reserved=0) requires authors to make all data underlying the findings described in their manuscript fully available without restriction, with rare exception (please refer to the Data Availability Statement in the manuscript PDF file). The data should be provided as part of the manuscript or its supporting information or deposited to a public repository. For example, in addition to summary statistics, the data points behind means, medians and variance measures should be available. If there are restrictions on publicly sharing data—e.g. participant privacy or use of data from a third party—those must be specified.

**Answer:** Thank you for your comment regarding data availability.

We confirm that the data underlying the findings of this study are available **upon reasonable request.** Due to institutional policies and patient privacy considerations, the raw data cannot be publicly shared in a repository. However, we are fully committed to transparency and will provide the **individual-level data points,** including those behind the reported means, medians, and variance measures, to researchers upon request.

This has been updated in the **Data Availability Statement** as follows:

**Data Availability Statement:** The data underlying this study cannot be made publicly available due to patient confidentiality restrictions. However, they will be made available to qualified researchers upon reasonable request. Please contact [Kwannapas_09@hotmai.com](mailto:Kwannapas_09@hotmai.com) for access and requests are subject to approval by the Faculty of Medicine, Chiang Mai University Ethics Committee.

Page 25 lines 417-420
